# Selective Pressure of Heavy Metals on Soil Microbial Taxa near a Smelting Area

**DOI:** 10.3390/toxics13121025

**Published:** 2025-11-27

**Authors:** Radina Nikolova, Evan Gatev, Anelia Kenarova, Michaella Petkova, Nikolai Dinev, Petr Baldrian, Galina Radeva

**Affiliations:** 1 Roumen Tsanev Institute of Molecular Biology, Bulgarian Academy of Sciences, Acad. G. Bonchev Str., Bl. 21, 1113 Sofia, Bulgaria; 2 Department of Ecology and Environmental Protection, Faculty of Biology, Sofia University “St. Kl. Ohridski”, 8 Dragan Tsankov Blvd., 1164 Sofia, Bulgaria; 3 N. Poushkarov Institute of Soil Science, Agrotechnologies and Plant Protection, Agricultural Academy, 7 Shosse Bankya St., 1331 Sofia, Bulgaria; 4 Laboratory of Environmental Microbiology, Institute of Microbiology of the Czech Academy of Sciences, Videnská 1083, 142 00 Prague 4, Czech Republic

**Keywords:** heavy metals, soil pollution, bacterial diversity, fungal diversity, bacterial resistance, metagenomics, functional profiling

## Abstract

Soil pollution by heavy metals (HMs) poses a major threat to soil quality and human health, with mining and smelting industries identified as key sources. Soils around smelters are often considered polluted hotspots, being generally unsuitable for agricultural activities. Although many studies have identified microbial taxa able to survive in such environments, most have focused on relatively low HM concentrations. The purpose of the study was to assess the ecological risk and to evaluate the diversity and structural shifts in microbial communities, as well as to predict key metabolic pathways associated with HM resistance in soils near Pb–Zn smelter in Bulgaria. The soils ranged from low-risk to disastrous, with cadmium (Cd) identified as the primary contributor to soil toxicity. High-throughput sequencing of 16S rRNA and ITS amplicons revealed widespread dominance of the phyla Proteobacteria, Actinobacteriota and Acidobacteriota, and Ascomycota, with the prevailing classes Acidobacteriae, Chloroflexia, and Eurotiomycetes, indicating their high tolerance to HMs. Functional predictions suggested enrichment of key pathways in the most polluted soils related to HM resistance, including efflux systems and detoxifying enzymes. These results highlight the necessity of integrating soil microbial indicators into agricultural management strategies to ensure safe food production.

## 1. Introduction

Soil pollution with HMs originating from anthropogenic activities, such as mining, smelting, foundries, and other industrial processes, poses a significant threat to soil quality and fertility, plant productivity, and human health [1]. Chronic exposure to elevated concentrations of HMs affects soil microorganisms, plants, and animals, both through direct contact with contaminated soils and via the trophic transfer of pollutants through food chains. Microbial communities, as vital components of soil, are essential to many ecological processes, including organic matter degradation and regulation of biogeochemical cycles, soil structure formation and stabilization, as well as suppression of plant pathogens [2]. Moreover, microbial communities respond immediately to HM stress, and their structural and functional responses make them reliable bioindicators of soil pollution and ecological disturbance [3]. Numerous studies have shown that HMs significantly reduce microbial diversity [4,5], richness [6], biomass and metabolic activity [5,6], and community structure and function [7,8,9].

The use of high-throughput sequencing technologies has significantly enhanced our understanding of microbial communities across various environments [10,11]. Metagenomic studies show that long-term HM exposure promotes tolerant taxa such as Proteobacteria, Actinobacteriota [6,12,13], Firmicutes [14], Gemmatimonadota and Chloroflexota [15,16]. It is also reported that the structure of soil fungal communities changes significantly depending on the level and type of HM pollution [17,18], and the dominant phyla in most of the studied soils are Ascomycota [19,20] and Zygomycota [21].

In general, microbial communities exposed to chronic HM pollution adapt by enhancing the activity of dominant and key taxa, reflecting their metabolic plasticity and functional redundancy [22,23]. Recent studies have shown that under HM stress, microbial community diversity and composition are primarily shaped by deterministic assembly processes [24,25]. The strong selective pressure from metal toxicity and altered soil properties favors taxa with specialized resistance traits, such as efflux pumps and detoxification enzymes [14,25,26].

In Bulgaria, heavy metals represent the primary pollutants affecting soil quality, due to intensive mining and smelting activities. The accumulation of HMs in agricultural soils, particularly in smelters’ vicinities, has become a pressing environmental issue, posing serious threats to food safety and human health. In the scientific literature, taxonomic shifts in microbial communities inhabiting HM polluted soils near smelters are well documented; however, most research has focused on (i) bacteria, with comparatively limited characterization of fungal communities and (ii) relatively low levels of HM pollution. Moreover, knowledge about the distribution of key pathways of HM resistance in multi-metal polluted soils remains incomplete. To bridge this gap, this study aims (i) to assess the ecological risk of soils for their inhabitants; (ii) to evaluate bacterial and fungal diversity and the shifts in their composition and structure across the HM pollution gradient, and (iii) to predict key metabolic pathways associated with HM resistance in bacterial communities. To achieve these aims, 16S rRNA and Internal Transcribed Spacer (ITS) ribosomal RNA sequencing were employed. The study focused on the area near the Pb-Zn smelter “KCM 2000”, located in the Plovdiv region of South-Central Bulgaria. Previous research has reported extremely high levels of soil polymetallic pollution (Pb, Zn, Cd, Cu, and As) in this region [27]. We hypothesized that HM pollution, as the primary factor, along with local soil properties, shaped the community structure and microbial distribution in long-term HM polluted soils, with key taxa maintaining stable and persistent profiles along the pollution gradient.

## 2. Materials and Methods

### 2.1. Study Area and Soil Sampling

**Figure 1:**
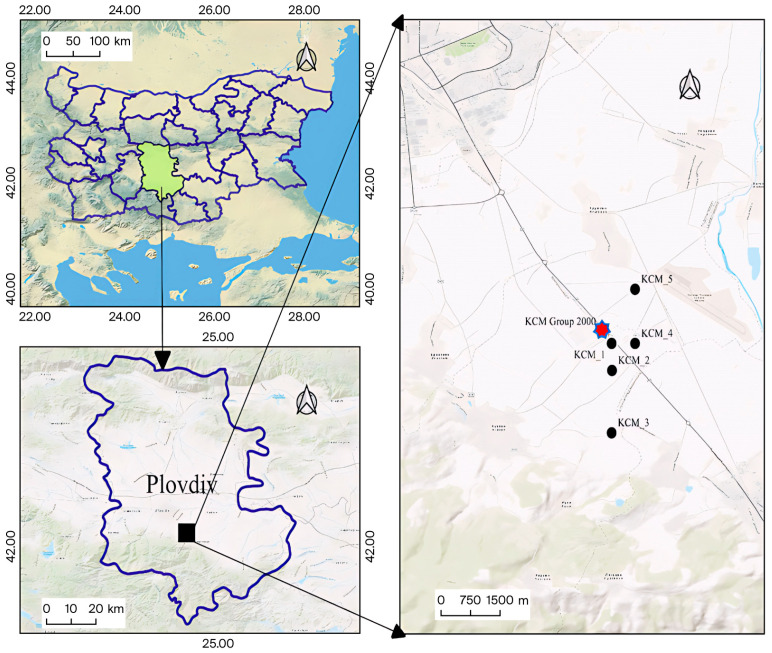
Map of sampling sites in the area of the “KCM 2000” smelter, Bulgaria.

The study area is located near the “KCM 2000” smelter (42°03′40.8″ N, 24°48′52.0″ E) (Figure 1) with more than 60 years of operational history. Long-term smelting activities have significantly impacted the local environment, particularly the soils, which are heavily polluted with Pb, Zn, Cd, Cu, and As [27].

Soil samples were collected in June 2020 from agricultural fields with industrial and oilseed crops (*Lavandula vera* L., *Gossypium* sp., *Brassica napus*). Five sampling sites were selected, depending on their distance from the source of pollution and in relation to previous data [27]. The sampling sites were as follows: KCM_1, located 0.5 km south of the smelter (42°03′31.68″ N, 24°49′19.2″ E); KCM_2, located 2 km south of the smelter (42°03′5.76″ N, 24°49′19.6″ E); KCM_3, located 3 km south of the smelter (42°02′6″ N, 24°49′19.2″ E); KCM_4, located 1 km southeast of the smelter (42°03′31.68″ N, 24°49′45.12″ E); KCM_5, located 1 km northeast of the smelter (42°04′23.52″ N, 24°49′45.12″ E). Soils were sandy loam textured with slightly acidic (KCM 4.1 and KCM_5) to neutral (KCM_1, KCM_2, KCM_3, KCM_4.2) pH and total organic carbon ranged from 7.04 g/kg (KCM_5.1) to 16.07 g/kg (KCM_4.2).

Five subsamples (200 g) were randomly collected at each site within a 100 m × 100 m quadrat from two soil depths: surface (0–20 cm) and subsurface (20–40 cm) layers. Post-collection, the soil samples were sieved, and a representative sample from each site and soil depth was prepared by mixing the subsamples into aliquots. The representative samples were divided into two portions: one was stored at −80 °C for molecular analysis, and the other at 4 °C for HM analyses.

### 2.2. Soil Concentrations of Heavy Metals and Ecological Risk Index

Heavy metal content in soils was measured as described in [8], and the concentrations of HMs are shown in Table S1. The potential ecological risk index (ERI) was calculated for both total and bioavailable forms of HMs according to [28]. Soil toxicity risk was assessed using the scale proposed by the same authors: ERI < 100 (low risk), 100 ≤ ERI < 150 (moderate risk), 150 ≤ ERI < 200 (considerable risk), 200 ≤ ERI < 300 (very high risk), and ERI ≥ 300 (disastrous risk). Due to the lack of relevant background data on unpolluted soil in the study area, the average of values below the maximum permissible concentrations (MPCs) specified by Bulgarian Regulation 3/2008 (https://www.moew.government.bg/bg/pochvi/zakonodatelstvo/nacionalno-zakonodatelstvo/ (accessed on 1 August 2008)) for each HM was used as a reference. Since the soil levels of Pb and Cd exceeded the MPCs, the respective MPC values were used in calculating the ERIs.

### 2.3. DNA Extraction and Illumina Sequencing

Total DNA was extracted from 0.5 g of soil using the E.Z.N.A. DNA Soil Kit (Omega Bio-tek, Norcross, GA, USA) following the manufacturer’s protocol. DNA concentration and purity were evaluated using a NanoDrop 1000 spectrophotometer (Thermo Scientific, Waltham, MA, USA) and verified by 1% agarose gel electrophoresis. Quantification was performed with a Qubit 4 Fluorometer (Thermo Scientific, Waltham, MA, USA). For bacterial community analysis, the hypervariable V3–V4 regions of the 16S rRNA gene were amplified using barcoded primers 341F (5′-CCTACGGGNGGCWGCAG-3′) and 806R (5′-GACTACHVGGGTATCTAATCC-3′) [29]. Amplicon libraries were prepared and sequenced on an Illumina MiSeq platform (Macrogen, Seoul, Republic of Korea) with a read length of 2 × 300 bp. Fungal ITS2 regions were amplified and sequenced following the methodology described by [30] in the Laboratory of Environmental Microbiology, Institute of Microbiology, Czech Academy of Sciences, Prague. This included PCR amplification with primers gITS7 (5′-GTGAATCATCGAATCTTTG-3′) and ITS4 (5′-TCCTCCGCTTATTGATATGC-3′), library preparation using the TruSeq PCR-Free Kit, (Illumina, San Diego, CA, USA) and sequencing on an Illumina MiSeq platform with a read length of 2 × 250 bp.

### 2.4. Bioinformatics and Data Processing

After trimming the barcodes, the raw sequencing data were processed using the standard QIIME2 pipeline [31]. Paired-end reads were denoised with DADA2 [32], applying truncation lengths of 225 and 300 bases for bacterial data and 173 and 131 bases for fungal data, based on read quality plots. Predicted functional changes in the bacterial community were assessed using the Phylogenetic Investigation of Communities by Reconstruction of Unobserved States (PICRUSt2) software package [33]. The complete PICRUSt2 pipeline, as implemented in the QIIME2 plug-in, was run on the denoised paired-end reads. The KO IDs obtained were manually annotated using the KEGG database to estimate HM resistance gene abundances.

Phylogenetic diversity analysis utilized trees generated with the “mafft” alignment method within QIIME2. Diversity metrics and group significance tests were calculated using the “diversity” plug-in library. The “core-metrics-phylogenetic” method was applied with a sampling depth of 12,650, determined by inspecting the feature table’s interactive sample detail. Taxonomic classification of bacterial sequences was performed using a Naive Bayes classifier pre-trained on the Greengenes 13_8 99% OTU reference database (2024.09.taxonomy) [34,35,36]. For fungal sequences, taxonomic classification was conducted using the UNITE 9 database [37]. OTUs were clustered at a 99% sequence identity threshold. Differential abundance testing between groups was performed using the Analysis of Composition of Microbiomes (ANCOM) method, implemented via the “q2-composition” plug-in, with pseudo-counts added to address zero frequencies. Chimera checking was conducted on aligned 16S rRNA and ITS sequences using the UCHIME algorithm [38].

### 2.5. Data Analyses

One-way ANOVA, followed by Tukey’s test, was performed to examine the significance of the differences in biotic and abiotic parameters.

The similarity of microbial communities was evaluated using a multi-dimensional scaling (NMDS) technique and taxonomic data on bacterial and fungal classes. NMDS was also used to visualize the distribution of KEGG pathways.

Two-way PERMANOVA, followed by SIMPER analyses, were conducted to assess the impact of ERI and soil depth on microbial distribution and identify the microbial classes most strongly contributing to intergroup differences in overall microbial community dissimilarity.

Pearson correlation analysis was performed to assess the relationships between ERI/soil depth, microbial classes and KEGG distributions.

Alpha diversity indices (Chao1, Shannon) were calculated based on bacterial and fungal taxonomic data.

The above statistics were performed with the PAST package [39] at a significance level of *p* < 0.05.

## 3. Results

### 3.1. Soil Concentrations of HMs and Ecological Risk Index

The HM pollution was severe, with mean soil concentrations recorded across soils as follows: As, 46.03 mg/kg; Cd, 46.87 mg/kg; Cu, 272.84 mg/kg; Pb, 2824 mg/kg; and Zn, 2991 mg/kg (Table S1). In most cases, these concentrations exceeded the MPC. In the context of total HM concentrations, ERIs were calculated (Table 1), and a gradient of HM ecological risk was established: Cd (disastrous) >> Pb (very high) >> As (moderate) >> Cu (low) > Zn (low).

The gradient of polymetallic ERI was arranged from the lowest to the highest risk as follows: KCM_3.2 < KCM_3.1 (low) << KCM_5.2 < KCM_5.1 (considerable) << KCM_2.1 < KCM_2.2 < KCM_4.2 < KCM_1.2 < KCM_4.1 < KCM_1.1 (disastrous). The difference in ERI between the surface- and subsurface soil layers was insignificant (F = 1.07; *p* = 0.47).

Additionally, bioavailable forms of Pb, Zn and Cd were measured in the surface soil layer, and their amounts varied, on average, from 0.03–0.08% (Pb and Zn) to 4.78% (Cd) from the respective total HM concentration (Table S2). The gradient of soil toxicity based on bioavailable forms of HMs arranged from the lowest to the highest ERI was as follows: KCM_2.1 << KCM_5.1 < KCM_3.1 << KCM_4.1 << KCM_1.1 (Table S2).

### 3.2. Taxonomic Composition and Structure of Soil Bacterial Communities

**Figure 2:**
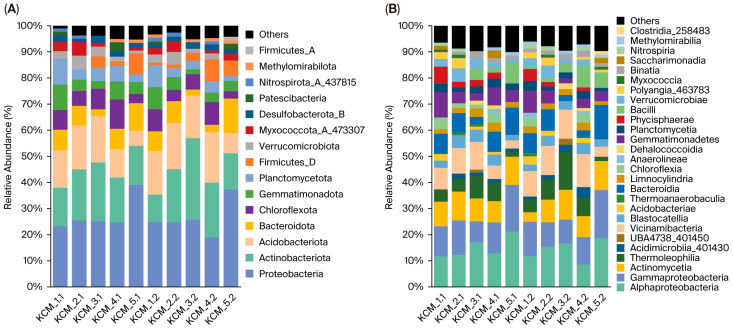
Relative abundance of bacterial phyla (**A**) and bacterial classes (**B**) in KCM soils. ‘Others’ represents a sum of relative abundances of phyla/classes < 1%.

Bacterial communities consisted of 15 phyla (Figure 2A). The dominant phylum was Proteobacteria, followed by Actinobacteriota and Acidobacteriota, which accounted for 26.96 ± 6.2%, 18.55 ± 5.8%, and 14.30 ± 4.1% of the total bacterial community, respectively. The subdominant phyla in soils were Bacteroidota, Chloroflexota, Gemmatimonadota and Planctomycetota, ranging from 4.87% to 7.06% of the total bacterial communities. Notably, Gemmatimonadota and Planctomycetota revealed a strong positive relationship with ERI (r ≥ 0.84; *p* = 0.002), representing up to 9.84% in the KCM_1.1 and up to 8.47% in the KCM_1.2 bacterial communities. Other predominantly local distributions were observed for Verrucomicrobiota and Myxococcota_A_473307 (KCM_2), Firmicutes_D (KCM_5), and Nitrospirota_A_437815 (KCM 1 and KCM_2). Low-abundant phyla Firmicutes_A and Methylomirabilota were represented in KCM_5.2 and all subsurface soils (except KCM_2.2), respectively. The effect of depth on the distribution of bacterial phyla was insignificant.

At the class level, taxonomic profiling of bacterial communities identified 27 classes (Figure 2B). The most widespread and dominant class across all soils was Alphaproteobacteria, averaging 14.66 ± 3.82%, followed by Gammaproteobacteria (12.30 ± 3.50%) and Actinomycetia (9.14 ± 2.33%). Subdominant classes in the bacterial community composition were Vicinamibacteria (8.53 ± 3.36%), Bacteroidia (6.99 ± 3.50%) and Thermoleophilia (6.35 ± 3.68%). Depth dependence, although insignificant in class distribution, was observed for Methylomirabilia, Acidimicrobiia_401430, Vicinamibacteria and Clostridia_258483 (r < 0.39; *p* > 0.26).

The diversity of bacteria at the class level (Table S3) was estimated by the following: (1) bacterial richness (Chao1), with a slightly, but not significantly higher number in the surface soil layer (492 ± 40) compared to the subsurface one (432 ± 90); (2) Shannon diversity index, with a higher but insignificantly different mean value in surface soils. The two indices showed significant correlation (F = 0.97, *p* < 0.0001), indicating the major role of taxonomic heterogeneity (Chao1) instead of class evenness in determining Shannon index values.

### 3.3. Taxonomic Composition and Structure of Soil Fungal Communities

**Figure 3:**
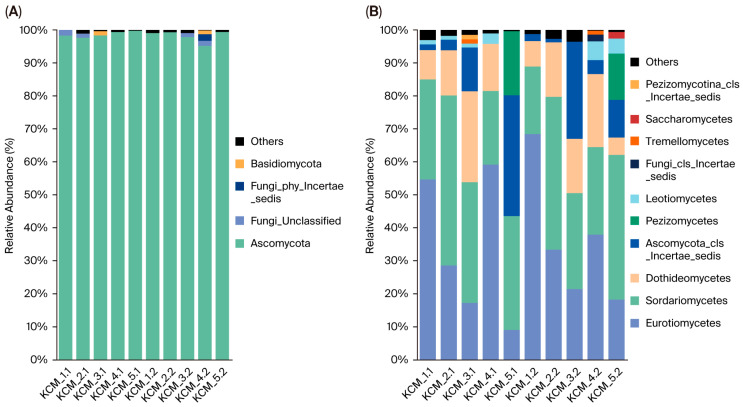
Relative abundance of (**A**) fungal phyla and (**B**) fungal classes in KCM soils. ‘Others’ represents a sum of relative abundances of phyla/classes < 1%.

The fungal communities were represented by 4 phyla (Figure 3A), with the dominance of Ascomycota, accounting for 95.20–99.79% of the fungal reads. Basidiomycota exhibited a local distribution, occurring in low abundances (<1.30%) in KCM_3. and KCM_4.2.

At the class level, 10 distinct fungal classes were identified in the studied soils (Figure 3B). Among these, Sordariomycetes, Eurotiomycetes and Dothideomycetes were almost universally distributed, except Dothideomycetes in KCM_5.1. Sordariomycetes and Eurotiomycetes can be considered dominant classes in fungal communities. The other two classes were considered as subdominants. A significant effect of soil depth was not recorded.

Fungal diversity across soils was estimated by Chao1 richness, ranging from 44 (KCM_1.1) to 118 (KCM_4.1), and Shannon index, ranging from 3.78 (KCM_1.1) to 5.99 (KCM_2.1) (Table S3). The surface soil layer showed higher values than the subsurface one, but the differences were insignificant (F ≤ 2.09; *p* ≥ 0.15).

### 3.4. Microbial Soil Similarity

**Figure 4:**
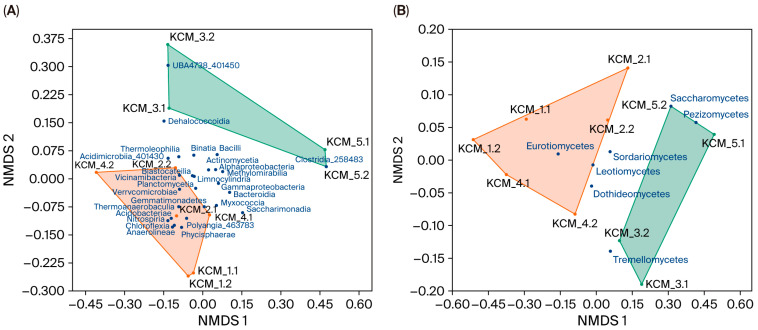
Non-metric multidimensional scaling (NMDS) plot of soil taxonomic similarity based on Euclidean resemblance matrix, calculated on (**A**) bacterial and (**B**) fungal classes. Two-dimensional stress: (**A**) 0.079 and (**B**) 0.088. Convex hulls of soils with ecological risk index (ERI) ≤ 181 and ERI ≥ 333 are shown in green and brown, respectively.

Non-metric multidimensional scaling (NMDS) was used to visualize the spatial arrangement of soils in ordination space, where the distances between points represented the class similarity of microbial communities (Figure 4). The clustering pattern of soils based on bacterial (Figure 4A) and fungal (Figure 4B) community compositions segregated the soils into two distinct groups: green colored with ERIs ≤ 180, and brown colored with ERIs ≥ 333. In both ordination plots, no overlapping of convex hulls was observed, indicating taxonomic differences between the soils. The NMDS analysis also revealed that some bacterial and fungal classes were distributed only in the convex hull of soils with low to considerable ecological risk (UBA4738_401450, Clostridia_258483, and Pezizomycetes), while others (Verrucomicrobiae, Thermoanaerobaculia, Chloroflexia and Eurotiomycetes) were associated with disastrous risk soils. A third group of microbial classes showed a more random distribution pattern, indicating a high tolerance to environmental factors, including HMs.

To evaluate the impact of potential soil toxicity (ERIs) and soil depth on microbial (bacterial and fungal) distribution, a two-way PERMANOVA was conducted (Table S4). PERMANOVA validated the NMDS ordination results, confirming the significant effect of soil toxicity (F ≥ 4.97; *p* < 0.002) on microbial distribution. In contrast, the effects of soil depth (F ≤ 0.69; *p* ≥ 0.62), and the interaction between soil depth and soil toxicity risk (F ≤ 0.82; *p* ≥ 0.48) were insignificant.

To identify the classes most strongly contributing to the differences in overall bacterial/fungal community dissimilarities, SIMPER analyses were conducted (Tables S5 and S6). The overall dissimilarity among bacterial communities was 31.79%, with six classes accounting for approximately 53% of this variation (Table S5). These classes were Alphaproteobacteria, Thermoleophilia, Gammaproteobacteria, Bacteroidia, Vicinamibacteria and Bacilli. Among these classes, Alphaproteobacteria (r = −0.57) and Bacilli (r = −0.36) showed strong but insignificant correlations with ERIs, while the relationships between other classes and soil toxicity risk were weak (r < −0.16) (Table S7). The overall average dissimilarity among fungal communities was 35.32% (Table S6) and Eurotiomycetes contributed most to this dissimilarity. Eurotiomycetes were associated with soils, having an ERI ≥ 333. Pearson correlation analysis confirmed strong correlations between ERI and the distribution of the class Eurotiomycetes (r = 0.73; *p* = 0.016) (Table S8).

### 3.5. Predicted Bacterial Resistance to Heavy Metals

Using the KEGG database, the functional pathways related to HM resistance of bacteria were identified among the total observed pathways (Table S9). A total of 26 dominant HM resistance pathways were selected, with a cumulative frequency of 717680. KEGG abundance was highest in KCM_1 (averaging 12.5%) and lowest in KCM_3 (averaging 7.9%). The most abundant pathway was K15726, followed by K07240 and K16264 (Table S10). Pathway K16264, associated with the genes *czc*D and *zit*B, which confer Zn-, Co-, and Cd-resistance, occurred at relatively high abundance across all soils. Genes involved in the HM efflux system czcA, *cus*A, and *cnr*A (K15726) were highly abundant in the severely polluted soils KCM_1 and KCM_2, and least abundant in the low-polluted soil KCM_3.2. All of these genes encode cation diffusion facilitators and efflux systems that transport bivalent metal ions out of the cytoplasm. Chromate efflux and reduction pathways K07240 and K19784, regulated by *chr*A and *chr*R, were detected in all soils, with K07240 showing high abundance and K19784 relatively low abundance. The Cu/Ag efflux pathways K07787 (*cus*A, *sil*A) and K07798 (*cus*B, *sil*B) were common across all soils, particularly in KCM_1 and KCM_2.1. The least abundant pathway, K12951 (*ctp*D), related to Co/Ni transport, was detected only in KCM_1. Additionally, less-distributed pathways were identified in KCM_3, including K00520, K08363, and K19058, all involved in bacterial resistance to Hg.

**Figure 5:**
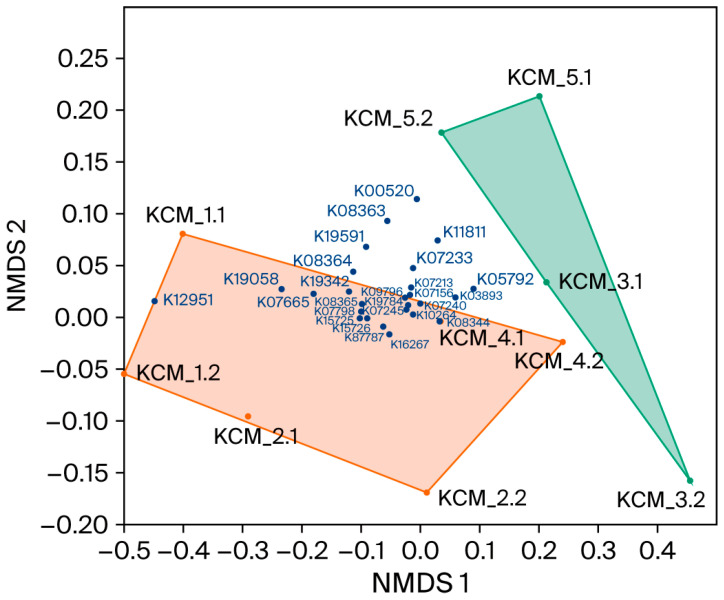
Non-metric multidimensional scaling (NMDS) plot of Kyoto Encyclopedia of Genes and Genomes (KEGG) pathways based on Euclidean resemblance matrix, calculated on bacterial 16S rRNA sequence data. Two-dimensional stress: 0.052. Convex hulls of soils with ecological risk index (ERI) ≤ 181 and ERI ≥ 333 are shown in green and brown, respectively.

To arrange the KEGG distribution across soils, NMDS was applied (Figure 5). NMDS technique ordinated 16 of 26 KEGG pathways in the convex hull of soils exhibiting disastrous risk (ERI ≥ 333), and none in the convex hull of soils with ERI ≤ 181. Some of the pathways were located outside of the two convex hulls, suggesting a relatively uniform distribution across soils.

Pearson correlation analysis confirmed the NMDS results, showing significant or insignificant but strong relationships between ERIs and KEGGs distribution (Table S10). Most correlations were positive, except for K05792, K03893, and K11811, which are pathways associated with bacterial resistance to the metalloids As and Te. The distribution of KEGGs across soil depth was insignificant and predominantly negative, although several relationships (K16264, K19784, K07156, and K07245) were notably strong.

## 4. Discussion

### 4.1. Potential Toxic Risk of Soil Environments

Soils in the vicinity of the “KCM 2000” smelter have been subject to HM pollution since the plant’s early operation. In the present study, the ecological risk index calculated from total HM concentrations ranged from low (KCM_3) to considerable (KCM_5) and disastrous (KCM_1, KCM_2, and KCM_4). Soils classified within the disastrous risk category exceeded the lower threshold of this range [28] by factors of two (KCM_2.2) to ten (KCM_1.1). Based on these results, the studied soils were categorized into two groups: (i) those with HM levels potentially tolerable by soil microorganisms without substantial alterations in community structure (KCM_3 and KCM_5), and (ii) those with pollution levels likely to induce significant shifts in microbial community composition and function (KCM_1, KCM_2, and KCM_4).

Additionally, the ecological risk was calculated based on bioavailable forms of HMs, and the values of ERIs classified the soils as low risk (KCM_2.1, KCM_3.1 and KCM_5.1), at the boundary between considerable and very high risk (KCM_4.1), and disastrous risk (KCM_1.1). It was reported [40] that metal toxicity is more closely related to the concentration of bioavailable forms than to total metal concentrations. On the other hand, the concentrations of bioavailable forms of HMs are much more variable than the total metal content and depend on metal speciation and the nature of its salts, as well as climatic and soil conditions [41,42]. Therefore, the bioavailable metal concentrations reported in the study are considered a snapshot of their possible soil levels, accounting for less than 0.24% of the total metal content, except for Cd, which ranges from 1.3% to 12.8%.

Cd and Pb in both forms (total and bioavailable) contributed the most to the calculated ERI values, owing to their high concentrations (bioavailable Cd and total Pb) and environmental toxicity (Cd) [21,43]. The average values of Cd’s single-factor environmental risk indices were 703 (total content) and 336 (bioavailable content), both of which fall under the category of disastrous environmental risk, making Cd the primary contributing factor to soil toxicity. In contrast, the single-factor environmental risk index for Pb was 141 for total content and 24 for bioavailable content, placing Pb in two distinct risk categories very high and low, respectively, depending on its chemical form. The low bioavailable risk level is attributed to Pb’s low water solubility [44], particularly in soils with neutral pH [45], which limits its mobility and microbial toxicity. Therefore, maintaining stable soil conditions, especially preventing acidification, is essential to avoid increased Pb solubilization and the consequent rise in its environmental risk.

### 4.2. Microbial Distribution

Long-term heavy metal (HM) pollution disrupts ecological balance and alters microbial community composition and function. This impact is particularly evident near “KCM 2000”—Plovdiv, where HM pollution remains severe and persistent. However, information on microbial community responses to such stress in this area is still limited. This study, therefore, examines taxonomic shifts in bacterial and fungal communities across different HM pollution levels and soil depths. Distribution of bacterial and fungal phyla across KCM soils harbors 15 bacterial and 4 fungal phyla. We assume that the high difference in the presence of phyla from the two microbial domains is largely related to inherent life strategies rather than higher fungal sensitivity to HMs. According to [46], bacteria, as r-strategists, can be represented by up to 8800 different species genomes, depending on soil type, while soil fungi, as K-strategists, form less diverse communities, occupying broader ecological niches (particularly trophic), and dominating in soil microhabitats in terms of biomass [47].

The bacterial communities were dominated by Proteobacteria, Actinobacteriota, and Acidobacteriota (Figure 2A), which were consistently identified as major phyla across all studied soils. Previous studies have similarly reported Proteobacteria, Acidobacteria, Actinobacteriota, and Chloroflexota as dominant taxa in smelting-polluted environments [48,49,50,51], while Firmicutes has been highlighted as one of the most resistant phylum in such environments [49]. The dominant phyla were also detected in both unpolluted soils [52,53] and HM polluted soils across various land uses, including agricultural fields [6,17,50,54] and areas affected by mining and industrial effluents [55,56]. Their widespread presence and persistence underscore their inherent tolerance and adaptability to HM stress, making them crucial components of microbial resilience in polluted ecosystems. Planctomycetota and Gemmatimonadota demonstrated higher relative abundance specifically for highly polluted soils KCM_1. It was also reported Planctomycetota and Gemmatimonadota are resistant to HMs and can survive in HM polluted soils [17].

Fungal KCM communities were dominated by Ascomycota. Several authors [57,58,59] have reported that Ascomycota colonize a wide range of environments, both disturbed and undisturbed. Moreover, Ascomycota was the highest relatively abundant fungus in HM polluted soils [17]. This adaptability of Ascomycota is attributed to a high number of genes associated with nutrition, carbohydrate metabolism, and a significantly high frequency of genomic traits linked to both stress tolerance and competitive abilities [57].

### 4.3. Distribution of Bacterial and Fungal Classes Across Soils

At the class level, bacterial richness and diversity were high and showed no significant differences between soils and soil depths. The equal bacterial distribution suggested that adaptation processes have occurred, likely selecting highly environmentally tolerant and flexible bacterial forms. The three most widespread bacterial phyla in KCM soils were represented by 10 of 27 identified classes. Alphaproteobacteria, Gammaproteobacteria, and Actinomycetia were identified as the dominant classes. High abundances of Alphaproteobacteria and Gammaproteobacteria have also been reported in smelting-polluted soils, along with Betaproteobacteria and Gemmatimonadetes [60,61]. Additionally, Thermoleophilia and Vicinamibacteria, Blastocatellia (Acidobacteriota), Bacteroidia (Bacteroidota), and Planctomycetia (Planctomycetota) were also relatively highly represented across soils.

All these classes were localized at an ordination plot (NMDS) between soil groups with low to considerable (KCM_3 and KCM_5) and disastrous (KCM_1, KCM_2, and KCM_4) ecological risks, and determined by SIMPER analysis as the main contributors to soil bacterial dissimilarity. Pearson correlation analysis confirmed the NMDS results, revealing weak relationships (either negative or positive) between bacteria from the aforementioned classes and ERIs, except for Alphaproteobacteria and Bacilli. The last two bacterial classes related strongly (although not statistically significant) and negatively to soil ecological risk, suggesting their preference to low-polluted soil habitats. For instance, [62] also found a reduction in Alphaproteobacteria abundance in agricultural soil polluted with Cd (1639 mg/ kg). In contrast, *Bacillus* sp. (class Bacilli) was highly abundant in Cd-polluted soils and could have potential for bioremediation in such sites [60]. Alphaproteobacteria and Bacilli were observed by other authors [63,64,65] as the most soil-abundant classes, which harbor a wide range of habitats, various kinds of metabolic processes, and cellular phenotypes. It includes mostly phototrophic genera, plant and animal pathogens, symbiotic relations with plants, phytohormone producers, etc.

Further, NMDS ordination positioned classes Chloroflexia (Chloroflexota), Gemmatimonadetes (Gemmatimonadota), Verrucomicrobiae (Verrucomicrobiota), Phycisphaerae (Planctomycetota), and Acidobacteriae (Acidobacteriota) within the convex hull of soils with disastrous ecological risk. All of these classes, except Gemmatimonadetes, showed significant positive correlations with ERIs and contributed approximately 4% to the inter-soil bacterial dissimilarity. Notably, the relative abundance of these classes increased in the most at-risk soil (KCM_1) compared to their mean abundances in the other soils. The increased abundances of certain bacterial taxa in highly HM-polluted soils have been reported by many authors [4,5,62], with these shifts in bacterial communities being attributed to the selectivity of HMs. The abundance of Verrucomicrobiae in KCM_1 was the same as the mean abundance for other soils. Based on the relatively even distribution of Verrucomicrobiae across soils and its negative correlation with ERI, we propose that this bacterial class should be classified alongside Alphaproteobacteria, Gammaproteobacteria, etc., rather than with the classes detected within the convex hull of disaster-risk soils. This class likely occupies a boundary position, as indicated in the ordination plot, where it is clearly situated between soils KCM_2.1 and KCM_2.2, which possessed the lowest ERI values compared to other soils in the disaster-risk category.

Finally, the distribution of certain bacterial classes (Acidimicrobiia_401430, Thermoanaerobaculia, and Binatia) in most soils, except for KCM_1 (ERI: 3636), suggests that representatives of these classes are generally HM-tolerant but not to the extreme pollution levels observed in KCM_1. On the contrary, the class UBA4738_401450, which was detected only in KCM_3 (low ecological risk), could be considered sensitive to HMs. However, the Pearson correlation analysis did not confirm these trends of distribution. All these bacterial classes correlated negatively with soil ecological risk, with the correlation for Acidimicrobiia_401430 (ERI_bioavailable HM_) and Binatia (both ERI_total HM_ and ERI_bioavailable HM_) being statistically significant. In this context, we assumed that soil toxicity non-linearly influenced bacterial distribution; likely this effect was modulated by local soil properties [66]. Our previous study [8] demonstrated the influence of soil pH, soil texture, and total organic carbon, along with HMs, on bacterial abundance and distribution in KCM soils. In general, in the context of observed trends in bacterial distribution, we propose the presence of three main groups of bacteria concerning soil toxicity and environmental stress: resistant (Chloroflexia, Gemmatimonadetes, Phycisphaerae and Acidobacteriae), tolerant (Alphaproteobacteria, Gammaproteobacteria, Actinomycetia, Verrucomicrobiae and many others), and sensitive (UBA4738_401450). The primary driving factors of their distribution were HM concentrations and local soil properties, but not soil depth. The Pearson correlations between bacterial distribution and soil depth were insignificant, with most being weak and negative.

The fungal diversity in KCM soils was represented by 10 classes, eight of which belong to the phylum Ascomycota. Fungal taxonomic richness and diversity were relatively lower compared to bacteria, but very similar to that shown in other studies [15]. The dominant classes in these soils included Eurotiomycetes and Sordariomycetes, while Dothideomycetes were classified as sub-dominants. Additionally, Pezizomycetes and Leotiomycetes (Ascomycota) were also widely distributed across the studied soils, but at low relative abundance. Tremellomycetes (Basidiomycota) were observed in very low abundances and showed localized distribution in KCM_2 and KCM_3, respectively.

NMDS ordination segregated the fungal classes Eurotiomycetes and Leotiomycetes within the convex hull of disastrous-risk soils, classifying them as resistant to HMs. Pearson correlation analysis further confirmed the positive correlations of these classes, particularly Eurotiomycetes, with the levels of soil toxicity. For example, Eurotiomycetes were present in low-risk soils of KCM_3 at 19.52%, in considerable-risk soils of KCM_5 at 13.64%, and in disastrous-risk soils at 46.44%. The resistance of Eurotiomycetes to HMs has been documented in early studies, where the authors demonstrated that their relative abundance in community structure increased with higher Cu and Cd [67], and Cd, Cr, Sn and Hg [68] pollution levels. Members of this class, such as *Aspergillus niger* and *Penicillium* sp., appear to evolve specific catabolic activities to use many pollutants as nutrients and energy sources [69]. For example, *Aspergillus* species show strong resistance to Cr and Pb through bioaccumulation and extracellular enzyme production, making them promising organisms for bioremediation [70].

The other groups of fungal classes segregated on the ordination plot included tolerant (Sordariomycetes, Dothideomycetes, and Tremellomycetes) and sensitive (Pezizomycetes and Saccharomycetes) classes. The abundances of these classes related negatively to HMs, which, although mostly strong, were statistically insignificant. The classes Pezizomycetes and Saccharomycetes had localized distribution in KCM_5. This distribution of Pezizomycetes confirmed the findings of [71] about the higher abundance of this class in soils with low concentrations of HMs. The class Sordariomycetes was evenly distributed across soils with varying ERI levels, ranging from 33.10% in low-risk soils to 39.01% in disastrous-risk soils. However, its highest relative abundance was observed in KCM_2 (average ERI: 504) and the lowest in KCM_1 (average ERI: 2422). This suggests that members of Sordariomycetes are more tolerant rather than resistant to HMs. Moreover, *Fusarium* species, which belong to this class, show strong biosorption and biotransformation abilities in Zn sequestration through organic acid production, intracellular compartmentalization, and enzymatic detoxification [70].

PERMANOVA identified HM concentrations as a driving factor of bacterial and fungal class segregation.

### 4.4. Distribution of HM Resistance Genes and Predicted Bacterial Resistance Pathways

In this study, the taxonomic distribution of bacteria in soils with varying ecological risk levels was analyzed alongside the distribution of genes encoding HM resistance. The results showed that pollution increased the frequency of HM resistance genes, with the highest frequency observed in the most ecological risk soils. This finding confirmed the results of earlier studies dedicated to HM toxicity and the distribution of potential functional pathways for resistance [72,73]. NMDS ordination revealed that approximately 62% of the dominant KEGG pathways were localized in disastrous-risk soils, demonstrating their role in adaptation to HM stress and resistance of bacteria. This was supported by the predominantly positive correlations between KEGG pathway abundances and ERIs. Among the 16 pathways linked to severe HM pollution, the most abundant were K12951, K15726, K15725, K07665, K07787, and K07798, which were associated with Cu, Co/Ni, and Cr efflux systems, which, after modification, could also be utilized by bacteria for defense against Zn, Cd, and Pb [74]. We suggest that KEGG pathways are related to the relatively abundance of Proteobacteria and classes Alphaproteobacteria and Gammaproteobacteria, which exhibit resistance to Cd and play important roles in Cd-contaminated soils [60,70]. The mechanism underlying Cd resistance is associated with the precipitation–dissolution balance, which constrains the dynamic fluctuations of free metal ions in the soil [75]. Our findings align with [62], who reported enrichment of such metabolic pathways in Cd-contaminated soils, indicating that microbial communities modify their metabolic processes to mitigate HM stress.

KEGG pathways positioned between the convex hulls on the NMDS plot were assigned with wider distribution and higher frequencies in lower- to considerable-risk soils compared to disastrous-risk soils. The primary defense mechanisms of these pathways for HM detoxification include enzyme activities, synthesis of periplasmic binding proteins, ion transport and ion efflux systems. Some of these defense pathways are widespread in protecting cells against excessive HM concentrations. It has been shown that HM resistance is the result of multiple layers of resistance systems with overlapping substrate specificities, but unique functions [74]. According to [74], some of these systems provide basic cellular defense against HMs, while others are highly specialized and found only in a few bacteria, conferring specific HM resistance. In general, predictive profiling identified efflux systems and detoxifying enzymes as prominent mechanisms for HM resistance (Tables S9 and S10). Among the metabolic pathways, those related to reducing the toxicity of Cr, Zn and Cd were most abundant, highlighting microbial ability to thrive in toxic environments by actively removing or immobilizing these metals.

## 5. Conclusions

The agricultural soils near the “KCM-2000” smelter were identified as risky from low to disastrous levels due to HM pollution, with Cd identified as the major contributor to toxicity. Microbial distribution patterns indicated the presence of HM-tolerant and HM-resistant bacterial and fungal phyla/classes. A strong positive correlation was observed between ERIs and the distribution of KEGGs, highlighting the adaptation that occurred in microbial communities according to the newly created soil environments. KEGG functional analysis revealed both widespread among soil locations common HM defense pathways and more complex mechanisms for HM-resistant bacteria in highly polluted soils. These results will be further validated in forthcoming studies. Overall, the findings highlight the substantial influence of the smelting industry on soil microbial diversity in surrounding areas and underscore the potential of microbially based approaches for monitoring soil health and promoting safe agricultural practices.

## Figures and Tables

**Table 1:** Ecological risk indices (ERIs) were calculated per HM and sampling site (total ERI).

Soil	Zn	Pb	Cd	Cu	As	Total ERI
KCM_1.1	40.55	578.45	2773.50	59.41	184.36	3636.27
KCM_2.1	6.68	68.51	235.50	9.26	12.93	332.88
KCM_3.1	0.93	6.78	58.50	3.58	9.27	79.05
KCM_4.1	29.48	286.15	1293.00	38.01	77.22	1723.86
KCM_5.1	3.17	16.75	139.50	6.54	14.96	180.93
KCM_1.2	14.83	187.20	927.00	22.07	58.49	1209.59
KCM_2.2	7.34	74.02	579.00	10.34	4.83	675.52
KCM_3.2	1.07	6.50	54.00	4.00	8.69	74.26
KCM_4.2	20.78	171.20	835.50	22.76	61.29	1111.53
KCM_5.2	3.47	16.50	135.00	5.88	12.26	173.11

## Data Availability

The datasets generated and analyzed during the current study are available in the NCBI Bioproject database under accession number PRJNA1363107.

## Supplementary Materials

The following supporting information can be downloaded at https://www.mdpi.com/article/10.3390/toxics13121025/s1, Table S1: Total concentrations of heavy metals/metalloids (HMs) in the surface and subsurface soils; Table S2: Water soluble concentrations of heavy metals (Pb, Zn, and Cd) in the surface soils, and their individual and total ecological risk index (ERI); Table S3: Diversity estimation statistics of microbial communities; Table S4: Results of the two-way PERMANOVA indicating the effects of soil toxicity (expressed as the Ecological Risk Index, ERI) and soil depth on the distribution of bacterial classes; Table S5: SIMPER results showing the contribution of bacterial classes to the total dissimilarity (31.79%) among soil bacterial communities; Table S6: SIMPER results showing the contribution of fungal classes to the total dissimilarity (42.03%) among soil fungal communities; Table S7: Pearson correlation coefficients between the relative abundances of bacterial classes, and soil toxicity (ERI) and depth (*n* = 10 for total HMs; n = 5 for bioavailable HMs). The level of significance is indicated in brackets. Significant correlations are bolted; Table S8: Pearson correlation coefficients between the relative abundances of bacterial classes, and soil toxicity (ERI) and depth (n = 10 for total HMs; n = 5 for bioavailable HMs). The level of significance is indicated in brackets. Significant correlations are bolted; Table S9: KEGG pathways providing bacterial heavy metal resistance in soils; Table S10: Definition of the KEGG pathways providing heavy metal resistance of bacteria in soils; Table S11: Pearson correlation analysis between the relative KEGG abundances, and soil risk of toxicity (ERI) and depth (n = 10 for total HMs; n = 5 for bioavailable HMs). The level of significance is indicated in brackets. Significant correlations are bolted.

## References

[1] Nyiramigisha P., Komariah, Sajidan. (2021). Harmful impacts of heavy metal contamination in the soil and crops grown around dumpsites. Rev. Agric. Sci..

[2] Campillo-Cora C., Rodríguez-Seijo A., Pérez-Rodríguez P., Fernández-Calviño D., Santás-Miguel V. (2025). Effect of heavy metal pollution on soil microorganisms: Influence of soil physicochemical properties. A systematic review. Eur. J. Soil Biol..

[3] Upadhyay V., Kumari A., Kumar S. (2024). From soil to health hazards: Heavy metals contamination in northern India and health risk assessment. Chemosphere.

[4] Chodak M., Gołębiewski M., Morawska-Płoskonka J., Kuduk K., Niklińska M. (2013). Diversity of microorganisms from forest soils differently polluted with heavy metals. Appl. Soil Ecol..

[5] Zampieri B.D.B., Pinto A.B., Schultz L., de Oliveira M.A., de Oliveira A.J.F.C. (2016). Diversity and distribution of heavy metal-resistant bacteria in polluted sediments of the Araça Bay, São Sebastião (SP), and the relationship between heavy metals and organic matter concentrations. Microb. Ecol..

[6] Cui H., Liu L.L., Dai J.R., Yu X.N., Guo X., Yi S.J., Zhou D.Y., Guo W.H., Du N. (2018). Bacterial community shaped by heavy metals and contributing to health risks in cornfields. Ecotoxicol. Environ. Saf..

[7] Hao J., Chai Y.N., Lopes L.D., Ordóñez R.A., Wright E.E., Archontoulis S., Schachtman D.P. (2021). The effects of soil depth on the structure of microbial communities in agricultural soils in Iowa (United States). Appl. Environ. Microbiol..

[8] Nikolova R., Petkova M., Dinev N., Kenarova A., Boteva S., Berov D., Radeva G. (2022). Correlation between bacterial abundance, soil properties and heavy metal contamination in the area of non-ferrous metal processing plant, Southern Bulgaria. BioRisk.

[9] Nikolova R., Kenarova A., Boteva S., Dinev N., Radeva G. (2024). Diversity and structure of soil bacterial communities in the area of non-ferrous metal plant revealed by 16S rRNA gene retrieval. C. R. Acad. Bulg. Sci..

[10] Coenen A.R., Hu S.K., Luo E., Muratore D., Weitz J.S. (2020). A Primer for Microbiome Time-Series Analysis. Front. Genet..

[11] Meng D., Li J., Liu T., Liu Y., Yan M., Hu J., Li X., Liu X., Liang Y., Liu H. (2019). Effects of redox potential on soil cadmium solubility: Insight into microbial community. J. Environ. Sci..

[12] Li J., Ma Y.B., Hu H.W., Wang J.T., Liu Y.R., He J.Z. (2015). Field-based evidence for consistent responses of bacterial communities to copper contamination in two contrasting agricultural soils. Front. Microbiol..

[13] Jiao S., Chen W., Wei G. (2019). Resilience and assemblage of soil microbiome in response to chemical contamination combined with plant growth. Appl. Environ. Microbiol..

[14] Wang Z., Deng G., Hu C., Hou X., Zhang X., Fan Z., Zhao Y., Peng M. (2025). Microbial diversity and community assembly in heavy metal-contaminated soils: Insights from selenium-impacted mining areas. Front. Microbiol..

[15] Pan X., Zhang S., Zhong Q., Gong G., Wang G., Guo X., Xu X. (2020). Effects of soil chemical properties and fractions of Pb, Cd, and Zn on bacterial and fungal communities. Sci. Total Environ..

[16] Liu N., Huang X., Sun L., Li S., Chen Y., Cao X., Wang W., Dai J., Rinnan R. (2020). Screening stably low cadmium and moderately high micronutrients wheat cultivars under three different agricultural environments of China. Chemosphere.

[17] Li J., Zheng Q., Liu J., Pei S., Yang Z., Chen R., Ma L., Niu J., Tian T. (2024). Bacterial-fungal interactions and response to heavy metal contamination of soil in agricultural areas. Front. Microbiol..

[18] Dusengemungu L., Gwanama C., Simuchimba G., Mubemba B. (2022). Potential of bioaugmentation of heavy metal-contaminated soils in the Zambian Copperbelt using autochthonous filamentous fungi. Front. Microbiol..

[19] Lin Y., Ye Y., Hu Y., Shi H. (2019). The variation in microbial community structure under different heavy metal contamination levels in paddy soils. Ecotoxicol. Environ. Saf..

[20] Chun S.J., Kim Y.J., Cui Y., Nam K.H. (2021). Ecological network analysis reveals distinctive microbial modules associated with heavy metal contamination of abandoned mine soils in Korea. Environ. Pollut..

[21] Zeng X.Y., Li S.W., Leng Y., Kang X.H. (2020). Structural and functional responses of bacterial and fungal communities to multiple heavy metal exposure in arid loess. Sci. Total Environ..

[22] Comte J., Fauteux L., del Giorgio P.A. (2013). Links between metabolic plasticity and functional redundancy in freshwater bacterioplankton communities. Front. Microbiol..

[23] Silva G.O.A., Southam G., Gagen E.J. (2023). Accelerating soil aggregate formation: A review on microbial processes as the critical step in a post-mining rehabilitation context. Soil Res..

[24] Chen Y., Tao S., Ma J., Qu Y., Sun Y., Wang M., Cai Y. (2024). New insights into assembly processes and driving factors of urban soil microbial community under environmental stress in Beijing. Sci. Total Environ..

[25] Sun T., Li G., Mazarji M., Delaplace P., Yang X., Zhang J., Pan J. (2024). Heavy metals drive microbial community assembly process in farmland with long-termbiosolids application. J. Hazard. Mater..

[26] Zhang M., Zhang T., Zhou L., Lou W., Zeng W., Liu T., Yin H., Liu H., Liu X., Mathivanan K. (2022). Soil microbial community assembly model in response to heavy metal pollution. Environ. Res..

[27] Yotova G., Padareva M., Hristova M., Astel A., Georgieva M., Dinev N., Tsakovski S. (2018). Establishment of geochemical background and threshold values for eight potential toxic elements in the Bulgarian soil quality monitoring network. Sci. Total Environ..

[28] Giri S., Singh A.K. (2017). Ecological and human health risk assessment of agricultural soils based on heavy metals in mining areas of Singhbhum copper belt, India. Hum. Ecol. Risk Assess. Int. J..

[29] Wen C., Wu L., Qin Y., Van Nostrand J.D., Ning D., Sun B., Xue K., Liu F., Deng Y., Liang Y. (2017). Evaluation of the reproducibility of amplicon sequencing with Illumina MiSeq platform. PLoS ONE.

[30] Bosch J., Némethová E., Tláskal V., Brabcová V., Baldrian P. (2023). Bacterial, but not fungal, communities show spatial heterogeneity in European beech (*Fagus sylvatica* L.) deadwood. FEMS Microbiol. Ecol..

[31] Bolyen E., Rideout J.R., Dillon M.R., Bokulich N.A., Abnet C.C., Al-Ghalith G.A., Alexander H., Alm E.J., Arumugam M., Asnicar F. (2019). Reproducible, interactive, scalable and extensible microbiome data science using QIIME 2. Nat. Biotechnol..

[32] Callahan B., McMurdie P., Rosen M., Han A., Johnson A., Holmes A. (2016). DADA2: High-resolution sample inference from Illumina amplicon data. Nat. Methods.

[33] Douglas G.M., Maffei V.J., Zaneveld J.R., Yurgel S., Brown J., Taylor C., Huttenhower C., Langille M. (2020). PICRUSt2 for prediction of metagenome functions. Nat. Biotechnol..

[34] McDonald D., Price M.N., Goodrich J., Nawrocki E.P., DeSantis T.Z., Probst A., Andersen G.L., Knight R., Hugenholtz P. (2012). An improved Greengenes taxonomy with explicit ranks for ecological and evolutionary analyses of bacteria and archaea. ISME J..

[35] McDonald D., Jiang Y., Balaban M., Cantrell K., Zhu Q., Gonzalez A., Morton J.T., Nicolaou G., Parks D.H., Karst S.M. (2024). Greengenes2 unifies microbial data in a single reference tree. Nat. Biotechnol..

[36] Robeson M.S., O’Rourke D.R., Kaehler B.D., Ziemski M., Dillon M.R., Foster J.T., Bokulich N.A. (2021). RESCRIPt: Reproducible sequence taxonomy reference database management. PLoS Comput. Biol..

[37] Nilsson R.H., Larsson K.H., Taylor A.F.S., Bengtsson-Palme J., Jeppesen T.S., Shigel D., Kennedy P., Picard K., Glöckner F.O., Tedersoo L. (2019). The UNITE database for molecular identification of fungi: Handling dark taxa and parallel taxonomic classifications. Nucleic Acids Res..

[38] Edgar R.C., Haas B.J., Clemente J.C., Quince C., Knight R. (2011). UCHIME improves sensitivity and speed of chimera detection. Bioinformatics.

[39] Hammer Ø., Harper D.A.T., Ryan P.D. (2001). PAST: Paleontological statistics software package for education and data analysis. Palaeontol. Electron..

[40] Kot A., Namieśnik J. (2000). The role of speciation in analytical chemistry. Trends Anal. Chem..

[41] Roane T.M., Josephson K.L., Pepper I.L. (2001). Dual-bioaugmentation strategy to enhance remediation of cocontaminated soil. Appl. Environ. Microbiol..

[42] Knotek-Smith H.M., Deobald L.A., Ederer M., Crawford D.L. (2003). Cadmium stress studies: Media development, enrichment, consortia analysis, and environmental relevance. Biometals.

[43] Håkanson L. (1980). An ecological risk index for aquatic pollution control—A sedimentological approach. Water Res..

[44] Welp G. (1999). Inhibitory effects of the total and water-soluble concentrations of nine different metals on the dehydrogenase activity of a loess soil. Biol. Fertil. Soils.

[45] Sjöstedt C., Löv Å., Olivecrona Z., Boye K., Kleja D.B. (2018). Improved geochemical modeling of lead solubility in contaminated soils by considering colloidal fractions and solid phase EXAFS speciation. Appl. Geochem..

[46] Torsvik V., Øvreås L. (2002). Microbial diversity and function in soil: From genes to ecosystems. Curr. Opin. Microbiol..

[47] Rajapaksha R.M.C.P., Tobor-Kapłon M.A., Bååth E. (2004). Metal toxicity affects fungal and bacterial activities in soil differently. Appl. Environ. Microbiol..

[48] Tipayno S.C., Truu J., Samaddar S., Truu M., Preem J.K., Oopkaup K., Espenberg M., Chatterjee P., Kang Y., Kim K. (2018). The bacterial community structure and functional profile in the heavy metal contaminated paddy soils, surrounding a nonferrous smelter in South Korea. Ecol. Evol..

[49] Fajardo C., Costa G., Nande M., Botías P., García-Cantalejo J., Martín M. (2019). Pb, Cd, and Zn soil contamination: Monitoring functional and structural impacts on the microbiome. Appl. Soil Ecol..

[50] Song J., Shen Q., Wang L., Qui G., Shi J., Xu J., Brookes P.h.C., Liu X. (2018). Effects of Cd, Cu, Zn and their combined action on microbial biomass and bacterial community structure. Environ. Pollut..

[51] Schneider A.R., Gommeaux M., Duclercq J., Fanin N., Conreux A., Alahmad A., Lacoux J., Roger D., Spicher F., Ponthieu M. (2017). Response of bacterial communities to Pb smelter pollution in contrasting soils. Sci. Total Environ..

[52] Kim H.S., Lee S.H., Jo H.Y., Finneran K.T., Kwon M.J. (2021). Diversity and composition of soil Acidobacteria and Proteobacteria communities as a bacterial indicator of past land-use change from forest to farmland. Sci. Total Environ..

[53] Mhete M., Eze P.N., Rahube T.O., Akinyemi F.O. (2020). Soil properties influence bacterial abundance and diversity under different land-use regimes in semi-arid environments. Sci. Afr..

[54] Wang X., Gao P., Li D., Liu J., Yang N., Gu W., He X., Tang W. (2019). Risk assessment for and microbial community changes in farmland soil contaminated with heavy metals and metalloids. Ecotoxicol. Environ. Saf..

[55] Beattie R.E., Henke W., Campa M.F., Hazen T.C., McAliley L.R., Campbell J.H. (2018). Variation in microbial community structure correlates with heavy-metal contamination in soils decades after mining ceased. Soil Biol. Biochem..

[56] Prakash A.A., Rajasekar A., Sarankumar R.K., AlSalhi M.S., Devanesan S., Aljaafreh M.J., Govarthanan M., Saye S.R.M. (2021). Metagenomic analysis of microbial community and its role in bioelectrokinetic remediation of tannery contaminated soil. J. Hazard. Mater..

[57] Egidi E., Delgado-Baquerizo M., Plett J.M., Wang J., Eldridge D.J., Bardgett R.D., Maestre F.D., Singh B.K. (2019). A few Ascomycota taxa dominate soil fungal communities worldwide. Nat. Commun..

[58] Passarini M.R.Z., Robayo M.I.G., Ottoni J.R., Duarte A.W.F.D., Rosa L.H. (2024). Biotechnological potential in agriculture of soil Antarctic microorganisms revealed by omics approach. World J. Microbiol. Biotechnol..

[59] Tedersoo L., Sánchez-Ramírez S., Kõljalg U., Bahram M., Döring M., Schigel D., May T., Ryberg M., Abarenkov K. (2018). High-level classification of the Fungi and a tool for evolutionary ecological analyses. Fungal Divers..

[60] Yu X., Zhao J., Liu X., Sun L., Tian J., Wu N. (2021). Cadmium Pollution Impact on the Bacterial Community Structure of Arable Soil and the Isolation of the Cadmium Resistant Bacteria. Front. Microbiol..

[61] Zou L., Lu Y., Dai Y., Khan M.I., Gustave W., Nie J., Liao Y., Tang X., Shi J., Xu J. (2021). Spatial variation in microbial community in response to As and Pb contamination in paddy soils near a Pb–Zn mining site. Front. Environ. Sci..

[62] Feng G., Xie T., Wang X., Bai J., Zhao H., Wei W., Wang M., Zhao Y. (2018). Metagenomic analysis of microbial community and function involved in Cd-contaminated soil. BMC Microbiol..

[63] Maheshwari P., Murali Sankar P. (2023). Culture-independent and culture-dependent approaches in symbiont analysis: In proteobacteria. Microbial Symbionts.

[64] Saxena A.K., Kumar M., Chakdar H., Anuroopa N., Bagyaraj D.J. (2019). Bacillus species in soil as a natural resource for plant health and nutrition. J. Appl. Microbiol..

[65] Wimalasekara R.L., Seneviratne K.N., Jayathilaka N. (2023). Metagenomics in bioremediation of metals for environmental cleanup. Metagenomics to Bioremediation.

[66] Guo H., Nasir M., Lv J., Dai Y., Gao J. (2017). Understanding the variation of microbial community in heavy metals contaminated soil using high throughput sequencing. Ecotoxicol. Environ. Saf..

[67] Guo Y., Cheng S., Fang H., Yang Y., Li Y., Zhou Y. (2022). Responses of soil fungal taxonomic attributes and enzyme activities to copper and cadmium co-contamination in paddy soils. Sci. Total Environ..

[68] Ye F., Gong D., Pang C., Luo J., Zeng X., Shang C. (2020). Analysis of fungal composition in mine-contaminated soils in Hechi City. Curr. Microbiol..

[69] Mohammadian E., Babai Ahari A., Arzanlou M., Oustan S., Khazaei S.H. (2017). Tolerance to heavy metals in filamentous fungi isolated from contaminated mining soils in the Zanjan Province, Iran. Chemosphere.

[70] Montes-Montes G., Munoz-Ramirez Z.Y., Cortes-Palacios L., Carrillo-Campos J., Ramirez-Sanchez O., Ortiz-Aguirre I., Munoz-Castellanos L.N., Gonzalez-Escobedo R. (2025). Microbial Diversity and Heavy Metal Resistome in Slag-Contaminated Soils from an Abandoned Smelter in Chihuahua, Mexico. Soil Syst..

[71] Lemmel F., Maunoury-Danger F., Leyval C., Cébron A. (2021). Altered fungal communities in contaminated soils from French industrial brownfields. J. Hazard. Mater..

[72] Chen J., Li J., Zhang H., Shi W., Liu Y. (2019). Bacterial heavy-metal and antibiotic resistance genes in a copper tailing dam area in northern China. Front. Microbiol..

[73] Goswami A., Adkins-Jablonsky S.J., Barreto Filho M.M., Shilling M.D., Dawson A., Heiser S., O’Connor A., Walker M., Roberts Q., Morris J.J. (2023). Heavy metal pollution impacts soil bacterial community structure and antimicrobial resistance at the Birmingham 35th Avenue Superfund site. Microbiol. Spectr..

[74] Nies D.H. (2003). Efflux-mediated heavy metal resistance in prokaryotes. FEMS Microbiol. Rev..

[75] Luo L., Xie L., Jin D., Mi B., Wang D., Li X., Dai X., Zou X., Zhang Z., Ma Y. (2019). Bacterial community response to cadmium contamination of agricultural paddy soil. Appl. Soil Ecol..

