# Supplementary material for: Selective Pressure of Heavy Metals on Soil Microbial Taxa near a Smelting Area
Source: Toxics. 2025 Nov 27;13(12):1025. doi: 10.3390/toxics13121025 (PMC12737216; doi:10.3390/toxics13121025)
Supplement: Supplementary file 1 [file toxics-13-01025-s001.zip › toxics-3973758-supplementary.pdf]

Supplemental Appendix

Supplement to: Selective Pressure of Heavy Metals on Soil Microbial Taxa Near a Smelting Area

Radina Nikolova, Evan Gatev, Anelia Kenarova, Michaella Petkova, Nikolai Dinev, Petr Baldrian, Galina Radeva

Supplemental data

Table S1. Total concentrations of heavy metals/metalloids (HMs) in the surface and sub-surface soils.

Soil	Zn	Pb	Cd	Cu	As
			(mg/kg)		
**KCM_1.1**	9452	11569	184.9	891.4	191.0
**KCM_2.1**	1558.2	1370	15.7	138.9	13.4
**KCM_3.1**	216.2	135.6	3.9	53.7	9.6
**KCM_4.1**	6872	5723	86.2	570.3	80.0
**KCM_5.1**	740.0	335.0	9.3	98.2	15.5
**KCM_1.2**	3457	3744	61.8	331.1	60.6
**KCM_2.2**	1712	1480	38.6	155.1	5.0
**KCM_3.2**	250.0	130.0	3.6	60.0	9.0
**KCM_4.2**	4843	3424	55.7	341.5	63.5
**KCM_5.2**	810.0	330.0	9.0	88.2	12.7
**MPC***	**320**	**100**	**2**	**150**	**25**

*MPC – Maximum permissible concentration allowed under Bulgarian Regulation 3/2008 (https://www.moew.government.bg/bg/pochvi/zakonodatelstvo/nacionalno-zakonodatelstvo/)

Table S2. Water soluble concentrations of heavy metals (Pb, Zn and Cd) in the surface soils, and their individual and total ecological risk index (ERI).

Soil	Heavy metal concentration (mg/kg)			Individual and total ERIs per HM and soil			
	Pb	Zn	Cd	Pb	Zn	Cd	Total
KCM_1.1.	2.6	8.2	9.0	65.0	82.0	1350	1497
KCM_2.1.	0.2	0.1	0.2	5.0	1.0	30.0	36.0
KCM_3.1.	0.9	0.1	0.5	22.5	1.0	75.0	98.5
KCM_4.1.	0.2	3.3	1.1	5.0	33.0	165.0	203.0
KCM_5.1.	0.8	0.3	0.4	20.0	3.0	60.0	83.0

Table S3. Diversity estimation statistics of microbial communities.

Soil sample	Bacterial diversity		Fungal diversity	
	Chao1	Shannon	Chao1	Shannon
**KCM_1.1.**	520	8.591	44	3.757
**KCM_2.1.**	466	8.417	117	5.991
**KCM_3.1.**	507	8.518	97	5.714
**KCM_4.1.**	531	8.468	118	4.836
**KCM_5.1.**	436	8.203	32	3.702
**KCM_1.2.**	557	8.625	60	4.072
**KCM_2.2.**	376	7.958	60	4.944
**KCM_3.2.**	324	7.916	50	4.637
**KCM_4.2.**	471	8.333	107	5.419
**KCM_5.2.**	452	8.312	47	4.274

Table S4. Results of the two-way PERMANOVA indicating the effects of soil toxicity (expressed as the Ecological Risk Index, ERI) and soil depth on the distribution of bacterial classes.

Source of variation	Sum of squares	Degrees of freedom	Mean square	F	p
**Bacterial communities**					
Ecological risk index (ERI)	609.18	2	304.59	4.97	0.002
Soil depth (D)	42.58	1	42.59	0.69	0.616
Interaction between ERI and D	72.16	2	36.08	0.59	0.822
Residuals	245.04	4	61.26		
Total	968.98	9			
**Fungal communities**					
Ecological risk index (ERI)	11579.7	2	5789.9	29.69	0.0001
Soil depth (D)	72.66	1	72.659	0.37	0.705
Interaction between ERI and D	321.94	2	160.97	0.82	0.482
Residuals	4679.56	24	194.98		
Total	16654	29			

Table S5. SIMPER results showing the contribution of bacterial classes to the total dissimilarity (31.79%) among soil bacterial communities

Class	Average dissimilarity	Individual contribution (%)	Cumulative contribution (%)
Alphaproteobacteria	3.18	10.02	10.02
Thermoleophilia	3.04	9.56	19.58
Gammaproteobacteria	2.98	9.39	28.96
Bacteroidia	2.91	9.15	38.11
Vicinamibacteria	2.49	7.82	45.93
Bacilli	2.18	6.86	52.79
Gemmatimonadetes	1.60	5.04	57.83
Actinomycetia	1.36	4.27	62.10
Phycisphaerae	1.36	4.27	66.37
Chloroflexia	1.25	3.92	70.28
Verrucomicrobiae	1.19	3.73	74.01
Polyangia_463783	0.96	3.03	77.04
Acidobacteriae	0.79	2.48	79.52
Acidimicrobiia_401430	0.68	2.14	81.66
Blastocatellia	0.64	2.01	83.67
Dehalococcoidia	0.59	1.84	85.51
UBA4738_401450	0.55	1.73	87.24
Nitrospiria	0.53	1.68	88.92
Binatia	0.53	1.67	90.60
Planctomycetia	0.53	1.66	92.26
Saccharimonadia	0.52	1.63	93.88
Limnocylindria	0.44	1.37	95.25
Methylomirabilia	0.37	1.17	96.42
Anaerolineae	0.36	1.13	97.55
Myxococcia	0.31	0.98	98.53
Thermoanaerobaculia	0.27	0.83	99.36
Clostridia_258483	0.20	0.64	100.00

Table S6. SIMPER results showing the contribution of fungal classes to the total dissimilarity (42.03%) among soil fungal communities

Class	Average dissimilaity	Individual contribution (%)	Cumulative contribution (%)
Eurotiomycetes	16.25	46.00	46.00
Sordariomycetes	8.52	24.12	70.11
Dothideomycetes	5.20	14.73	84.84
Pezizomycetes	3.74	10.59	95.44
Leotiomycetes	1.19	3.37	98.81
Tremellomycetes	0.22	0.62	99.42
Saccharomycetes	0.20	0.58	100.00

Table S7. Pearson correlation coefficients between the relative abundances of bacterial classes, and soil toxicity (ERI) and depth (n=10 for total HMs; n=5 for bioavailable HMs). The level of significance is indicated in brackets. Significant correlations are bolted.

	$ERI_{total\ HM}$	$ERI_{bioavailable\ HM}$	Soil depth
$ERI_{total\ HM}$	1.00 (0.00)	**0.93 (0.021)**	-0.26 (0.47)
$ERI_{bioavailable\ HM}$	**0.93 (0.021)**	1.00 (0.00)	
Soil depth	-0.26 (0.47)	-	1.00 (0.00)
Alphaproteobacteria	-0.57 (0.09)	-0.48 (0.41)	-0.12 (0.75)
Gammaproteobacteria	-0.14 (0.70)	-0.17 (0.78)	-0.05 (0.89)
Actinomycetia	-0.31 (0.38)	-0.18 (0.77)	-0.23 (0.52)
Thermoleophilia	-0.16 (0.67)	-0.15 (0.81)	0.23 (0.52)
Acidimicrobiia_401430	-0.31 (0.38)	**-0.94 (0.018)**	0.38 (0.28)
UBA4738_401450	-0.37 (0.29)	-0.25 (0.67)	0.16 (0.66)
Vicinamibacteria	0.00 (0.99)	0.11 (0.85)	0.36 (0.30)
Blastocatellia	-0.19 (0.59)	-0.45 (0.44)	-0.22 (0.54)
Acidobacteriae	**0.64 (0.04)**	**0.88 (0.038)**	0.21 (0.55)
Thermoanaerobaculia	-0.20 (0.59)	-0.46 (0.43)	-0.14 (0.70)
Bacteroidia	0.05 (0.88)	0.10 (0.88)	-0.06 (0.87)
Limnocylindria	-0.06 (0.88)	-0.49 (0.40)	-0.18 (0.63)
Chloroflexia	**0.82 (0.004)**	**0.76 (0.041)**	-0.27 (0.46)
Anaerolineae	0.28 (0.44)	-0.16 (0.79)	0.17 (0.64)
Dehalococcoidia	-0.21 (0.56)	-0.34 (0.50)	0.06 (0.86)
Gemmatimonadetes	**0.85 (0.002)**	**0.88 (0.038)**	-0.24 (0.56)
Planctomycetia	0.58 (0.08)	0.55 (0.33)	-0.22 (0.53)
Phycisphaerae	**0.83 (0.003)**	**0.91 (0.023)**	-0.29 (0.42)
Bacilli	-0.36 (0.30)	-0.36 (0.55)	0.17 (0.65)
Verrucomicrobiae	0.08 (0.82)	-0.06 (0.92)	-0.20 (0.59)
Polyangia_463783	0.27 (0.44)	0.33 (0.58)	0.07 (0.84)
Myxococcia	0.16 (0.65)	0.43 (0.47)	-0.03 (0.93)
Binatia	**-0.67 (0.034)**	**-0.77 (0.039)**	0.22 (0.54)
Saccharimonadia	0.43 (0.21)	0.31 (0.60)	-0.23 (0.52)
Nitrospiria	0.40 (0.25)	0.56 (0.32)	0.27 (0.45)
Methylomirabilia	-0.21 (0.55)	-0.35 (0.55)	0.40 (0.26)
Clostridia_258483	-0.24 (0.51)	-	0.33 (0.35)

Table S8. Pearson correlation coefficients between the relative abundances of bacterial classes, and soil toxicity (ERI) and depth (n=10 for total HMs; n=5 for bioavailable HMs). The level of significance is indicated in brackets. Significant correlations are bolted.

	$ERI_{total\ HM}$	$ERI_{bioavailable\ HM}$	Soil depth
$ERI_{total\ HM}$	1.00 (0.00)	**0.93 (0.021)**	-0.26 (0.47)
$ERI_{bioavailable\ HM}$	**0.93 (0.021)**	1.00 (0.00)	0.00 (1.00)
Soil depth	-0.26 (0.47)	0.00 (1.00)	1.00 (0.00)
Eurotiomycetes	**0.73 (0.02)**	0.60 (0.29)	0.06 (0.87)
Sordariomycetes	-0.42 (0.22)	-0.32 (0.52)	-0.08 (0.83)
Dothideomycetes	-0.15 (0.69)	-0.21 (0.74)	0.05 (0.90)
Pezizomycetes	-0.35 (0.33)	-0.27 (0.86)	-0.08 (0.93)
Leotiomycetes	0.10 (0.78)	0.06 (0.92)	0.17 (0.64)
Tremellomycetes	-0.17 (0.64)	-0.25 (0.68)	-0.03 (0.93)
Saccharomycetes	-0.24 (0.51)	0.00 (1.00)	0.33 (0.35)

Table S9. KEGG pathways providing bacterial heavy metal resistance in soils

KEGGs	Soil KEGG abundances									
	**KCM_1.1**	**KCM_1.2**	**KCM_2.1**	**KCM_2.2**	**KCM_3.1**	**KCM_3.2**	**KCM_4.1**	**KCM_4.2**	**KCM_5.1**	**KCM_5.2**
K16264	8132	8543	9042	7043	8604	7142	7334	7850	8016	7840
K16267	2490	2906	2716	1953	2309	1618	1887	2471	1068	1249
K15725	8434	9346	8069	5636	4370	2595	5763	4384	4271	5417
K15726	14466	15794	13352	10697	8217	5238	9869	7732	7716	9851
K07240	9411	10141	9114	7656	10807	7536	9283	11076	11489	10663
K19784	3567	3119	2885	2791	2903	2485	3258	2160	3246	3578
K12951	64	59	0	0	0	0	0	0	0	0
K07787	5790	7000	7078	5415	4894	3310	3859	4705	3612	4392
K07798	5177	6458	5321	3774	2495	1523	3524	2867	3565	4296
K19591	135	245	119	49	36	59	64	0	156	505
K05792	25	5	311	138	174	120	129	353	403	215
K00520	116	63	149	68	0	3	61	22	420	538
K08363	337	36	194	63	61	0	137	24	389	418
K08364	594	347	332	202	101	49	135	183	371	511
K08365	2089	1963	1322	1059	1071	565	1299	712	980	1523
K19058	884	667	225	164	57	0	338	35	228	245
K03893	1553	1109	1916	1225	2688	2189	1558	2619	2889	2367
K11811	570	664	758	242	875	668	660	205	1962	2488
K07156	3652	2676	2818	2083	2743	2165	2432	2425	3728	3399
K07213	4056	3202	2864	2037	3444	2103	2519	2988	4486	3964
K07233	1107	1108	1199	755	870	892	759	388	1892	2755
K07245	4731	3584	3986	3068	3515	2951	3404	3582	3797	3485
K07665	5073	4066	1942	1380	693	282	2551	582	1808	2016
K08344	338	228	295	283	441	444	206	186	377	165
K09796	4817	5233	4645	3540	4255	3280	4450	3199	6017	5425
K19342	1752	1672	1229	870	509	252	917	466	1160	1452

Table S10. Definition of the KEGG pathways providing heavy metal resistance of bacteria in soils

KEGG symbol	Definition
K16264	Cobal-zinc-cadmium efflux system protein (czcD, zitB)
K16267	Zinc and cadmium transporter (zipB)
K15725	Outer membrane protein, heavy metal efflux system (czcC, cusC, cnrC)
K15726	Heavy metal efflux system protein (czcA, cusA, cnrA)
K07240	Chromate transporter (chrA)
K19784	Chromate reductase, NAD(P)H dehydrogenase (quinone) (chrR, NQR)
K12951	Cobalt/nickel-transporting P-type ATPase D (ctpD)
K07787	Copper/silver efflux system protein (cusA, silA)
K07798	Membrane fusion protein, copper/silver efflux system (cusB, silB)
K19591	MerR family transcriptional regulator, copper efflux regulator (cueR)
K05792	Tellurite resistance protein (terA)
K00520	Mercuric reductase (merA)
K08363	Mercuric ion transport protein (merT)
K08364	Periplasmic mercuric ion binding protein (merP)
K08365	MerR family transcriptional regulator, mercuric resistance operon regulatory protein (merR)
K19058	Mercuric ion transport protein (merC)
K03893	Arsenical pump membrane protein (arsB)
K11811	Arsenical resistance protein (arsH)
K07156	Copper resistance protein C (copC, pcoC)
K07213	Copper chaperone (copZ, golB, ATOX1, ATX1)
K07233	Copper resistance protein B (pcoB, copB)
K07245	Copper resistance protein D (pcoD)
K07665	Two-component system, OmpR family, copper resistance phosphate regulon response regulator CusR (CusR, copR, silR)
K08344	Suppressor for copper-sensitivity B (scsB)
K09796	Periplasmic copper chaperone A (pccA)
K19342	Copper chaperone (nosL)

Table S11. Pearson correlation analysis between the relative KEGG abundances, and soil risk of toxicity (ERI) and depth (n=10 for total HMs; n=5 for bioavailable HMs). The level of significance is indicated in brackets. Significant correlations are bolted.

	$ERI_{total\ HM}$	$ERI_{bioavailable\ HM}$	Soil depth
$ERI_{total\ HM}$	1.00 (0.00)	0.93 (0.021)	-0.257 (0.47)
$ERI_{bioavailable\ HM}$	0.93 (0.021)	1.00 (0.00)	-
Soil depth	-0.257 (0.47)	-	1.00 (0.00)
K16264	-0.41 (0.49)	-0.17 (0.79)	-0.44 (0.21)
K16267	0.28 (0.64)	0.31 (0.61)	-0.05 (0.89)
K15725	0.63 (0.25)	0.61 (0.28)	-0.17 (0.63)
K15726	0.66 (0.22)	0.66 (0.22)	-0.14 (0.71)
K07240	-0.54 (0.35)	-0.34 (0.58)	-0.23 (0.52)
K19784	**0.86 (0.039)**	**0.81 (0.049)**	-0.40 (0.25)
K12951	**0.90 (0.034)**	**1.00 (0.00)**	-0.02 (0.95)
K07787	0.12 (0.85)	0.23 (0.71)	-0.03 (0.92)
K07798	0.49 (0.40)	0.50 (0.39)	-0.08 (0.82)
K19591	0.21 (0.73)	0.32 (0.60)	0.25 (0.49)
K05792	**-0.81 (0.049)**	-0.73 (0.15)	-0.17 (0.64)
K00520	-0.24 (0.69)	-0.15 (0.81)	-0.03 (0.93)
K08363	0.32 (0.59)	0.43 (0.46)	-0.38 (0.28)
K08364	0.62 (0.26)	0.76 (0.13)	-0.14 (0.69)
K08365	**0.94 (0.019)**	**0.94 (0.015)**	-0.20 (0.57)
K19058	**0.96 (0.008)**	**0.96 (0.009)**	-0.23 (0.58)
K03893	-0.76 (0.13)	-0.54 (0.34)	-0.18 (0.61)
K11811	-0.54 (0.35)	-0.41 (0.49)	-0.08 (0.82)
K07156	0.28 (0.64)	0.51 (0.38)	-0.47 (0.17)
K07213	0.09 (0.88)	0.36 (0.54)	-0.39 (0.26)
K07233	-0.29 (0.63)	-0.12 (0.84)	0.01 (0.98)
K07245	0.70 (0.19)	**0.86 (0.044)**	-0.58 (0.07)
K07665	**0.96 (0.010)**	**0.92 (0.024)**	-0.26 (0.47)
K08344	-0.35 (0.56)	-0.01 (0.98)	-0.36 (0.30)
K09796	-0.15 (0.81)	-0.04 (0.94)	-0.39 (0.26)
K19342	0.72 (0.17)	0.76 ().13)	-0.17 (0.63)
